# Prevalence of wasting and associated factors among children aged 6–59 months in Habro district, Eastern Ethiopia: a cross-sectional study

**DOI:** 10.3389/fnut.2024.1353086

**Published:** 2024-07-05

**Authors:** Sileshi Tilahun, Kedir Teji Roba, Hirbo Shore Roba, Jemal Ahmed Nure, Teshome Sosengo, Behailu Hawulte Ayele, Melat B. Maruta, Adera Debella, Addis Eyeberu, Ibsa Mussa

**Affiliations:** ^1^Habro Woreda Health Office, West Hararghe, Oromia, Ethiopia; ^2^School of Public Health, College of Health and Medical Sciences, Haramaya University, Harar, Ethiopia; ^3^School of Pharmacy, College of Health and Medical Sciences, Haramaya University, Harar, Ethiopia; ^4^Obstetrics and Gynecology, Menelik Hospital, Addis Abeba, Ethiopia; ^5^School of Nursing and Midwifery, College of Health and Medical Sciences, Haramaya University, Harar, Ethiopia

**Keywords:** wasting, children, outpatient, discharge, Ethiopia

## Abstract

**Background:**

Globally, five million children under the age of five died in 2021. Asia and African countries contributed to 69% and 27.2% of wasting, respectively. In Ethiopia, out of 901 (10.1%) under-five children, 632 (8.1%) were found to be moderately wasted, and 269 (3.0%) were severely wasted. The purpose of this study was to assess the prevalence of wasting and its associated factors among children between the ages of 6 and 59 months in Habro Woreda, Oromia, Eastern Ethiopia.

**Methods:**

A community-based cross-sectional study was conducted in Habro Woreda from 25 August to 20 September 2020. In total, 306 participants were included in this study through a systematic sampling technique. Data were collected using a pretested structured questionnaire through a face-to-face interview, entered into EpiData version 3.1, and analyzed using SPSS version 25. Predictors were assessed using a multivariate logistic regression analysis model and reported using an adjusted odds ratio (AOR) with a 95% confidence interval (CI). Statistical significance was set at *p* < 0.05.

**Results:**

Overall, the prevalence of wasting among children aged 6–59 months in the Habro district was 28%, with a 95% confidence interval [26.5, 32.2%]. Factors such as mothers illiterate [AOR = 3.4; 95% CI: 1.14–10.47], households without latrines [AOR = 2.91; 95% CI: 1.33–6.37], food-insecure households [AOR = 4.11; 95% CI: 1.87–9], households that did not receive home visits [AOR = 4.2; 95% CI: 1.92–9.15], did not eat a variety of food [AOR = 7.44; 95% CI: 2.58–21.45], sick children after discharge from the program [AOR = 6.55; 95% CI: 2.85–15.02], readmitted children [AOR = 3.98; 95% CI: 1.43–15.07], and wasting 3.42 [AOR = 3.42; 95% CI: 1.24–9.45] were factors statistically associated with outcome variables.

**Conclusion:**

This study noted that the prevalence of wasting among children aged 6–59 months following discharge from the Outpatient Therapeutic Program remains high. Educational status of the mother, availability of a latrine, separate kitchen in the household, household food insecurity, household dietary diversity, home visit, and admission type were significantly associated with wasting of children after discharge from the outpatient therapeutic program. Therefore, efforts that target these factors should be maximized to reduce the occurrence of wasting among children aged 6–59 months after discharge from the outpatient therapeutic program.

## 1 Introduction

Many economically disadvantaged countries struggle with malnutrition as a serious public health issue (1). Malnutrition exists either in the form of undernutrition or overnutrition. Inadequate intake of carbohydrates, protein, vitamins, and mineral supply to the cells of the body to satisfy the physiological requirements are the immediate causes of undernutrition (2–4). In developing countries, poverty and high food costs are major causes of undernutrition (5). Malnutrition is classified into two types: acute (wasting) and chronic (6, 7).

Malnutrition results in over two million deaths of children under 5 years annually (8). Worldwide, socio-demographic, childcare, environmental health, and sanitation risk variables have a significant impact on the prevalence of wasting among children under 5 years (9–13). Evidence-based interventions (i.e., interventions targeting pathways of wasting in life cycles, including *in-utero* environment, the early period of infancy, and a focus on the process and identification of wasting rather than the state of being wasted) are recommended to overcome the problem of wasting (14, 15).

In 2022, an estimated 45 million children under five (6.8%) were affected by wasting; out of which, 13.6 million (2.1%) were severely wasted (16). More than three-quarters of all children with severe wasting live in Asia, and another 22% live in Africa (13). A study in Ethiopia found that out of 901 children (10.1%) under five 632 (8.1%) were moderately wasted, and 269 (3.0%) were severely wasted (17). According to the Ethiopia Mini-Demographic and Health Survey (EMDHS), 7.2% of children in Ethiopia were wasted (18). The survey indicated the regional variations in the percentage prevalence of wasting in under-five children: 21% in Somalia, 14% in Afar, 13% in Gambella, 2% in Addis Ababa, and 4% in Harari (18), 14.7% in Wolkite town (19), 13.4% in Bulehora district (20), 10% in Northwest Ethiopia (21), 11% in Adi-Harush and Hitsats Refugee Camps (22), and 7.2% in Wukro town (9) Tigray Region, 11.1% in Dilla town (10), and 28.2% in Hawassa Zuria district (11).

Despite the commitment of various stakeholders and the fact that the government of Ethiopia has already achieved remarkable progress in reducing under-five mortality in the last decades and designed multi-sectorial approaches to address malnutrition, the prevalence of wasting and factors influencing wasting in children aged between 6–59 months remain unabated in the study area (12, 23, 24). To the best of our knowledge, documentation of evidence regarding wasting at the country level and the study area is lacking. Therefore, this study aimed to assess the prevalence of wasting and associated factors among children aged between 6 and 59 months in Habro Woreda, Oromia, East Ethiopia.

## 2 Materials and methods

### 2.1 Study design, setting, and period

A community-based cross-sectional study was conducted from 25 August to 20 September 2020, among children aged 6–59 months, 6 months after discharge from an outpatient therapeutic program in Habro Woreda, Oromia, Eastern Ethiopia. Habro Woreda is located about 320 km east of Addis Ababa, the capital city of Ethiopia. Habro Woreda has 1 general hospital, 7 type B functional health centers, and 32 functional health posts. In 2020, there were approximately 44,240 under-five children and 55,765 households in the region, with a total population size of 269,272. According to a projection by the Ethiopian Central Statistical Agency in 2011, the population was 190,455 (17). With a mostly rural population (88%), the area is characterized by a high population density (242/km^2^), fragmented farmland ownership, and limited income-generating opportunities. However, the major source of income was cash crops, mainly focused on chat cultivating and chat trading. We used the strengthening the reporting of observational studies in epidemiology (STROBE) cross-sectional checklist for this study (25).

### 2.2 Population and eligibility criteria

All children aged 6–59 months who lived in the Habro district served as the study population. Mothers who had resided for at least 6 months in the study area and had a child aged 6–59 months were included. However, children with evidence of physical impairments, serious illness or mental impairments, children whose names differed from those present on the outpatient therapeutic program registration logbook, children who had left the selected study area, and mothers or caregivers who were unable to communicate were excluded from the study.

### 2.3 Data collection methods and procedures

A questionnaire was developed after reviewing related studies in different literature, with some questions adopted from the related studies and contextualized to align with the study objectives and data extraction form accordingly. Data were collected using a pretested and structured interviewer-administered questionnaire to collect data on socio-demographic characteristics, environmental hygiene and sanitation characteristics, and childcare practice and health characteristics, which comprised the three survey components. The wording and sequence of questions were designed in such a way that the sequence of ideas (from general to specific and from easy to difficult questions) was maintained. Standard anthropometric measurements were taken from all subjects during data collection. We use the Waterlow height-for-age (%) method of classification (26). Length was measured for children who were < 87 cm or those too weak to stand in supine (lying down). However, if the children were more than 87 cm in height, their height was recorded using a stadiometer to the nearest 1 cm. To ensure the quality of data, seven skilled health professionals who had completed BSc Nursing and one supervisor who was proficient in both Afaan Oromo and Amharic collected the data, and the task was closely supervised by the investigators. Data collectors and supervisors received 4 days of training in the data-gathering processes. Initially, we translated the questions while keeping the purpose of the questionnaire and the intent of the questions in mind. It was translated by group members who were bilingual experts. To ensure the accuracy of the translation, the questionnaire was translated back into English by someone who had not seen the original version and was unfamiliar with the questionnaire's context. The back-translated version was then compared with the original, and any differences in meaning were corrected. Then, to ensure cross-validity, we attempted to interview a set of respondents in English and another set in the local language, such as Afaan Oromo and Amharic; their responses were then compared to identify differences in understanding. Finally, pretesting was conducted to identify questions that were poorly understood, ambiguous, or elicited hostile or other undesirable responses. We attempted to conduct a pretest using the already translated questionnaire. We tried to implement all the steps in pretesting, such as obtaining an evaluation of a questionnaire and testing the revised questionnaire through its paces with friends and colleagues. We statistically calculated Cronbach's alpha, which is a measure used to assess the quality of the instruments we used. The outcome was 0.87, which was within acceptable ranges.

### 2.4 Variables and their measurement

#### 2.4.1 Dependent variable

Wasting (Yes/No).

#### 2.4.2 Independent variables

**Socio-demographic variables:** Child age, child sex, maternal educational status, maternal occupation, family size, religion, ethnicity, and household food security.

**Environmental health-related variables:** Availability of latrine, hand washing practice, solid waste disposal, and availability of liquid waste disposal pit.

**Child caring practice and health characteristics:** Exclusive breastfeeding, dietary diversity score, meal frequency, vaccination status, history of diarrhea in the past 2 weeks, ever used family planning, and place of delivery.

### 2.5 Operational definitions

**Wasting:** Wasting is defined as the child's weight-for-height Z-score (WHZ) < -2 SD from the median WHO reference values; WHZ ≥-3SD and < -2SD indicates moderate wasting; and WHZ < -3SD indicates severe wasting (19, 27).

**Undernutrition:** A mid-upper arm circumference **(**MUAC) below 12.5 cm indicates acute undernutrition; MUAC ≥ 11.5 cm and < 12.5 cm indicates moderate acute undernutrition; and MUAC < 11.5 cm indicates severe acute undernutrition (19, 27).

**Household food security:** It is measured by whether the respondent worries that the household is lacking enough food for the past 4 weeks (4, 19).

**Food secure:** If the respondent does not worry about the household lacking enough food for the past few weeks (4, 19).

**Food insecurity:** The lack of regular access to enough safe and nutritious food for normal growth and development and active and healthy life. This may be due to the unavailability of food and/or a lack of resources to obtain food (4, 19, 28).

**Mildly food insecure:** It is when a respondent rarely worries about food (once or twice in the past 4 weeks) (4, 19, 28).

**Moderately food insecure**: It is when a respondent sometimes worries about food (3 to 10 times in the past 4 weeks) (4, 19, 28).

**Severely food insecure**: It is when a respondent often worries about food (more than 10 times in the past 4 weeks) (4, 19, 28).

**Household dietary diversity**: Food intake that includes all of the diversified dietary needs of the organism in the correct proportion (29).

**Diarrhea:** It is defined as having three or more loose, watery stools in 24 h in the 2 weeks before the survey (30).

**Improved drinking water**: Drinking water that is obtained from piped water into dwellings, public taps, protected dug wells, protected springs, and rainwater (29).

**Wasting**: A nutritional deficiency condition of recent onset related to sudden food deprivation or malabsorption of nutrients, resulting in weight loss and weight-for-height below −2 SD, but resolved upon treatment (31).

**Moderate wasting:** Weight-for-height below −2 Z-score of children under 5 years of age (32).

**Severe wasting**: Weight-for-height or length < -3 Z-score of children under 5 years of age (31).

### 2.6 Bias

There were a number of biases involved while conducting this research, and the researcher took explicit measures to avoid them. One of the biases was social desirability and recall bias, and to avoid it, the researcher paraphrased the questions in a way that was not socially desirable and made an attempt to provide a clue for the intended question that needed to be answered.

### 2.7 Sample size determination

The sample size was determined by using the single population proportion formula, considering the following assumptions:


$$\begin{array}{l} \frac{\left(Z\alpha /2\right)2\;p\;\left(1-p\right)}{d2} \end{array}$$

where n = minimum sample size required for the study, Z = standard normal distribution (Z = 1.96), with a confidence interval of 95% and α= 0.05, p = proportion of wasting taken from proportion of wasting taken from the Ethiopian Demographic Health Survey, which is 10% (33), d = maximum acceptable margin of sampling error (d) = 5% = 0.05, and design effect = 1.5. Therefore, the final total sample size, including the contingency (10%) of the non-response rate, was 188. The sample size for the second objective (factors) was also determined by using the double population proportion formula for a cross-sectional study by considering the following assumptions: power = 80%, CI = 95%, and ratio = 1:1. The largest sample size from those samples was taken and the final sample size was calculated to be 306 children paired with their mothers or caregivers.

### 2.8 Sampling procedures

In this study, a three-stage sampling strategy was followed. In the first stage, four health centers and three health posts were randomly selected and included. The number of study units to be sampled from each health facility was determined using proportional allocation to size based on the number of client flows in the current year (2019), ensuring equal weighting despite varying numbers of health centers and posts (34). Children aged 6–59 months who had recovered from the OTP program (November 2019–February 2020) were recruited from the OTP registration logbook. Then, the systematic random sampling method was employed to select each study subject. The sampling fraction of the Kth interval was calculated as (N/n = 640/306 = 2.1~.2). Based on the client's registration logbook number, the starting participant was determined by using the lottery method, and every second discharged child was included until the required sample was achieved (33). The eligible participants who did not make themselves available during the data collection period in the selected Kebele were revisited up to three times, and if they did not make themselves available after three visits, the data collector skipped that household and interviewed the next household as a substitute. Finally, a total of 306 study samples were selected using a systematic sampling procedure. Then, each patient was given a unique code.

### 2.9 Data quality control

A multilingual specialist (Afaan Oromo and Amharic) created the questionnaire first in English and then translated it into the local languages. To maintain uniformity, it was then translated back into English. Data collectors and supervisor were trained on how to use the data-gathering instrument and processes. A pretest was conducted with 5% of the research participants under comparable conditions prior to the real data collection. Regular supervision was provided by investigators and competent research supervisors.

### 2.10 Methods of data analysis

First, the obtained data were reviewed for completeness and consistency. They were then cleaned, coded, and entered into EpiData version 3.1 for further analysis. The entered data was transferred to SPSS version 25 for analysis. Descriptive and summary statistics were collected and reported using frequency tables and figures. A binary logistic regression model was constructed to test for a connection between independent factors and the outcome variable. Hosmer–Lemeshow statistics and Omnibus tests were used to evaluate the model's fitness. To determine the genuine determinants of the outcome variables, a multivariable analysis was performed. Using the standard error and co-linearity statistics, a multi-collinearity test was performed to assess for the presence of correlation between independent variables, and no collinearity effects were observed. As a result, the variance inflation factor (VIF) was 0.951. The odds ratio (OR) and 95% confidence interval (CI) were used to determine the direction and degree of the statistical connection. In all bivariable and multivariable analyses, a *p*-value of 0.05 was considered statistically significant.

## 3 Results

### 3.1 Socio-demographic characteristics

In this study, a total of 306 children aged 6–59 months were included. Out of the total study participants, 156 (51%) were boys, and the mean age of the study children and their mothers was 30 (SD+6.5) months and 29.4 (SD+10.8) years, respectively. Regarding religion, most of the caregivers 278 (90.8%) were Muslim. Of the participants, 138 (45.1%) mothers and caregivers of children belong to an age group of 21–29 years. With regard to educational status, most of the mothers or caregivers 217 (70.9%) were illiterate. The majority 288 (94.1) of the caregivers were housewives (Table 1).

**Table 1:** Socio-demographic characteristics associated with children aged 6–59 months in Habro Woreda, East Ethiopia 2020 (n = 306).

**Variable**	**Category**	**Frequency**	**Percentage (%)**
Sex of the child	Male	156	51.0
	Female	150	49.0
Child age	12–24 months	124	40.5
	25–36 months	112	36.6
	37–48 months	53	17.3
	49–60 months	17	5.6
Gender of the household head	Male	277	90.5
	Female	29	9.5
Age of mothers/caregivers	≤ 20yrs	16	5.3
	21–29 years	138	45.1
	30–39 years	128	41.8
	≥40 years	24	7.8
Religion	Muslim	278	90.8
	Orthodox	26	8.5
	Protestant	2	0.7
Educational status of the mother	Illiterate	217	70.9
	Literate	89	29.1
Occupation of mother/caregiver	Housewife	288	94.1
	Government worker	3	1.0
	Merchant	15	4.9
Family size	≤ 3	30	9.8
	4–6	171	55.9
	≥7	105	34.3
Amount of farmland owned	No farmland	13	4.2
	1–4 qindi^*^ of farmland	160	52.3
	>4 qindi of farmland	133	43.5
Source of income from farmland	Only khat	10	3.4
	Only corn	42	14.3
	Both khat and corn	207	70.6
	Both vegetables and sweet potatoes	11	3.8
	Mixed farming	23	7.9
Wealth quintiles	Low	107	35.0
	Middle	98	32.0
	High	101	33.0

^*^1-hectar = 4–qindi.

### 3.2 Environmental hygiene and sanitation characteristics

Among the total households, 195 (63.7%) had a latrine, and of those households that had a latrine, only 19 (9.7%) of them had a handwashing facility. A total of 215 (70.3%) households had access to improved sources of drinking water, 209 (68.3%) households lacked proper waste disposal facilities, and 39 (12.7%) households had no separate kitchens (Table 2).

**Table 2:** Environmental- and sanitation-related associated with children aged 6–59 months after 6 months following discharge from OTP in Habro Woreda, East Ethiopia 2020 (n = 306).

**Variables**	**Category**	**Frequency**	**Percent**
Availability of latrine	No	111	36.3
	Yes	195	63.7
Latrine with hand washing	No	176	90.3
	Yes	19	9.7
Waste disposal	Improper	209	68.3
	Proper	97	31.7
Source of drinking water	Improved	215	70.3
	Unimproved	91	29.7
Separate kitchen	No	39	12.7
	Yes	267	87.3

### 3.3 Child caring practice and health characteristics

#### 3.3.1 Morbidity status of the children

Among the children, 134 (28.4%) had a history of illness, and of these, 56 (64.4%), 21 (24.1%), and 10 (11.5%) had diarrhea, pneumonia, and fever, respectively. Among those children with diarrhea, about 9 (2.9%) did not get oral rehydration solution (ORS) or zinc. Furthermore, about 126 (41.2%) of children did not take vitamin A supplementation within the last 6 months after being discharged from OTP. In their lifetime, 123 (40.2%) of the children had not received the measles vaccine (Table 3).

**Table 3:** Treatment outcomes of children aged 6–59 months after 6 months following discharge from OTP in Habro Woreda, East Ethiopia 2020 (n = 306).

**Variables**	**Category**	**Frequency**	**Percent (%)**
Child illness	Yes	134	43.8
	No	172	56.2
Diarrhea	Yes	56	64.4
Pneumonia	Yes	21	24.1
Fever	Yes	10	11.5
Oral rehydration solution (ORS) and zinc	Yes	9	16.1
	No	47	83.9
Finished zinc	Yes	19	40.4
	No	28	59.6
Vit A supplementation	No	126	41.2
	Yes	180	58.8
Measles immunization	No	123	40.2
	Yes	183	59.8

#### 3.3.2 Family substance abuse

Of the total respondents, 257 (84.0%) of the study participants' household family members chewed khat, with 215 (83.7%) of them chewed khat daily. Of the total households, 212 (69.3%) of children's family members smoked cigarettes, and among these, 82 (59.6%) were heavy smokers (Table 4).

**Table 4:** Substance abuse among family members of the children aged 6–59 months after 6 months of discharge from OTP in Habro Woreda, East Ethiopia 2020 (n = 306).

**Variables**	**Category**	**Frequency**	**Percentage (%)**
Family members who chew khat	No	49	16.0
	Yes	257	48.0
How frequently do they chew khat	Every day	215	83.7
	Every other day	26	10.1
	Only on weekend	16	6.2
Any family member smoking a cigarette	Yes	212	69.3
	No	94	30.9
How frequently do they smoke	Heavy smoker	82	38.7
	Moderate smoker	105	49.5
	Light smoker	25	11.8

#### 3.3.3 Dietary diversity score of households

**Figure 1:**
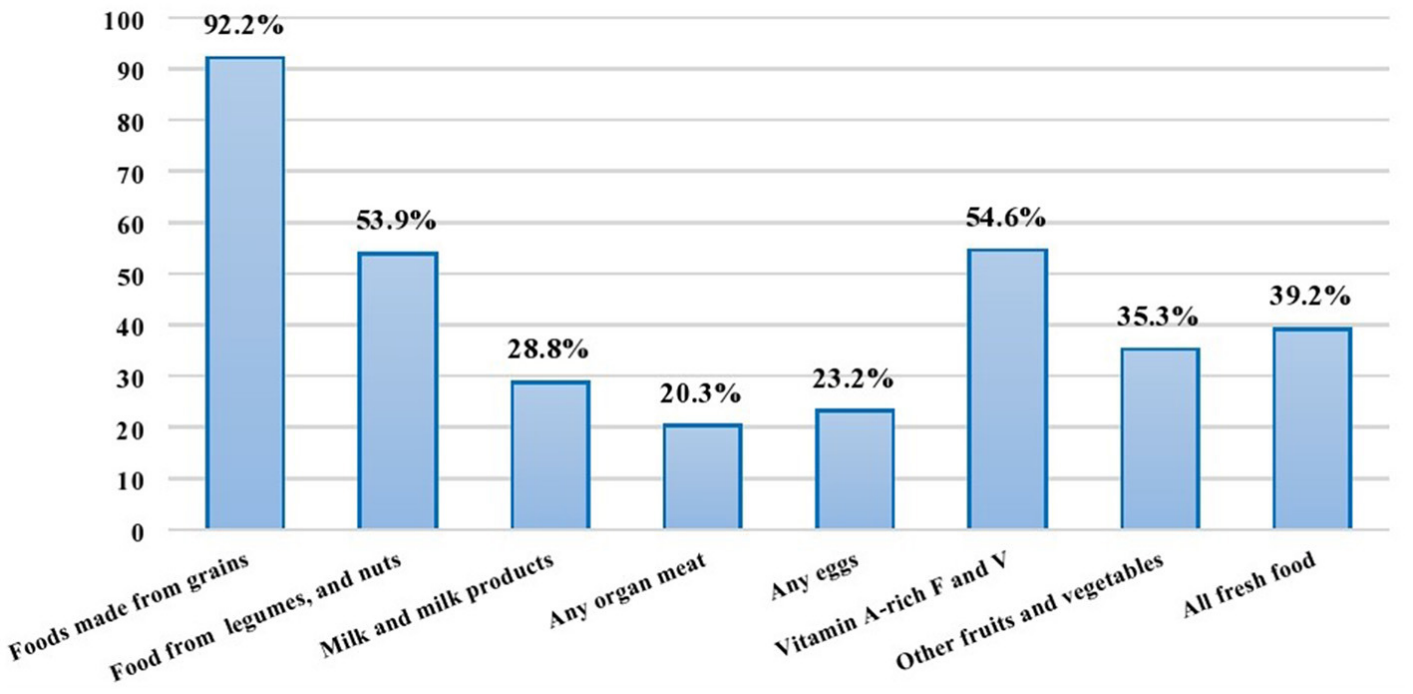
Food groups consumed by children aged 6–59 months in Habro Woreda, Eastern Ethiopia, 2020 (*n* = 306).

According to this study, 92.2% of children consumed foods made from grains, roots, and tuber products; 54.6% consumed foods rich in vitamin A-rich fruits and vegetables; 53.9% consumed foods made from pulses, legumes, and nuts; 35.3% consumed other fruits and vegetables; and 28.8% consumed milk and dairy products (Figure 1).

#### 3.3.4 Level of food insecurity

**Figure 2:**
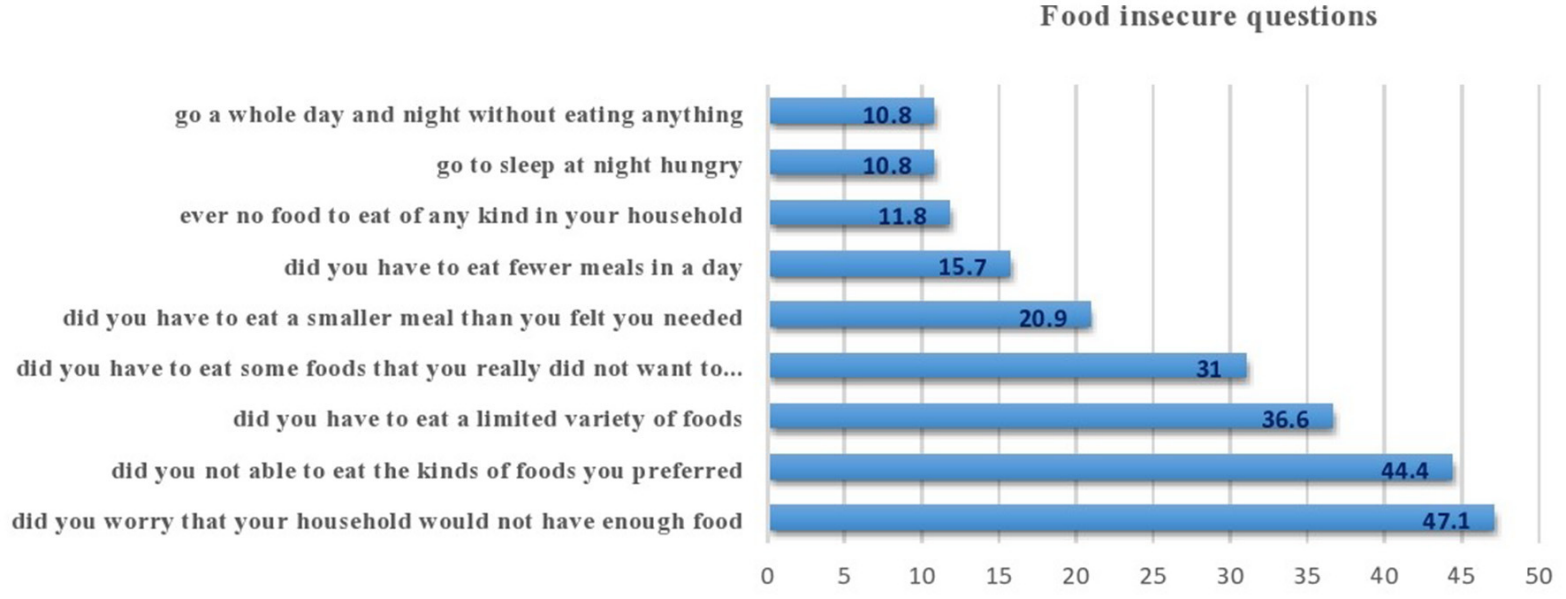
Status of food insecurity among children aged 6–59 months in Habro Woreda, Eastern Ethiopia, 2020 (*n* = 306).

According to the household food insecurity access scale, 138 households (45.1%) were food insecure, whereas 168 households (54.9%) were food secure. Nearly 63 (45.7%) reported concern about not having enough food to eat in the household, and 75 (54.3%) reported that household members went to bed hungry (Figure 2).

#### 3.3.5 Anthropometric status of children

The prevalence of wasting among children aged 6–59 months following exit from the outpatient therapeutic program within the last 11 months was 28.0%. At admission, 272 (88.9%) of the children were newly admitted to OPT. Out of them, 183 (59.8%) children did not receive a home visit or follow-up by health workers within the last 11 months following exit from an outpatient program (Table 5).

**Table 5:** Anthropometric status of children aged 6–59 months after 6 months following exit from OTP in Habro Woreda, East Ethiopia 2020 (n = 306).

**Variables**	**Category**	**Frequency**	**Percent (%)**
Category of wasting	Normal	216	70.6
	Wasted	90	29.4
Bilateral pitting edema	Yes	6	2.0
	No	300	98.0
Follow-up after discharge	Yes	123	40.2
	No	183	59.8
Type of admission	New	272	88.9
	Readmission	34	11.1

#### 3.3.6 Distribution of wasting among the study participants

**Figure 3:**
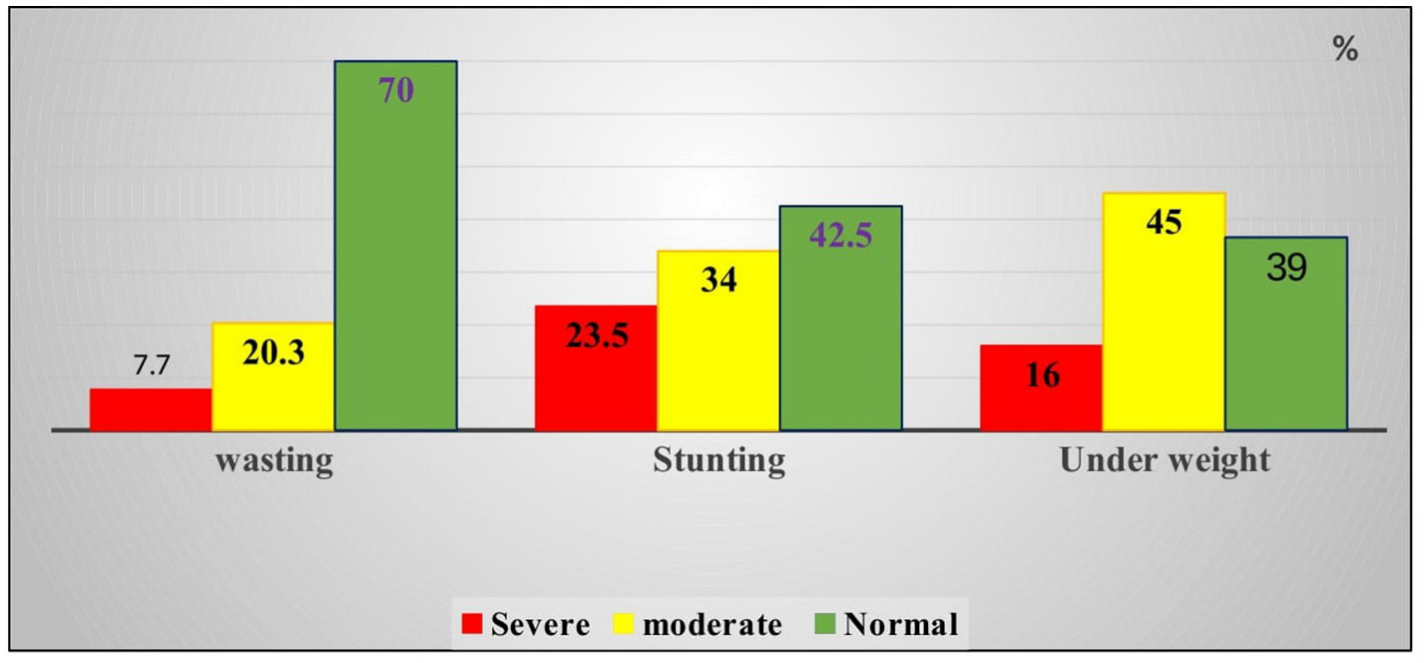
Percentage of wasting, stunting, and underweight among children aged 6–59 months in Habro Woreda, East Ethiopia, 2020 (*n* = 300 for wasting and underweight, *n* = 306 for stunting).

The prevalence of wasting among children aged 6–59 months following exit from the outpatient therapeutic program within the last 11 months was 28.0% (95% CI: 26.5–32.2%). Of these, 7.7% was severely wasted and 20.3% was moderately wasted (Figure 3).

#### 3.3.7 Wasting and its associated factors

First, bivariate analysis was conducted, and variables with a *p*-value of < 0.25 were included in multivariate analysis. In the multivariate analysis, variables with a *p-*value of < 0.05 were declared statistically significant predictors of wasting. Variables such as the gender of the household head, educational status of the mother, availability of latrine, source of drinking water, separate kitchen, smoking, household food insecurity, household dietary diversity, home visit, child illness, vitamin A supply for the last 6 months, and admission type were significantly associated with child wasting in the bivariate binary logistic regression. However, in the multivariable logistic regression, factors such as the educational status of the mother, availability of a latrine, separate kitchen, household food insecurity, household dietary diversity, home visit, and admission type were significantly associated with wasting.

Accordingly, children born to mothers who were illiterate were 3.4 [AOR = 3.4; 95% CI: 1.14–10.47] times more likely to be wasted as compared to those whose mothers who were literate. Similarly, children from households without latrines were 2.9 [AOR = 2.91; 95% CI: 1.33–6.37] times more likely to encounter wasting than their counterparts. The households that do not have separate kitchens were 3.42 [AOR = 3.42; 95% CI: 1.24, 9.45] times more likely to encounter waste than their counterparts. Similarly, the odds of experiencing wasting among children from food-insecure households were 4.11 [AOR = 4.11; 95% CI: 1.87–9] times higher than those of their counterparts.

Moreover, the odds of household dietary diversity were 7.44 [AOR =7.44; 95% CI: 2.58, 21.45] times higher among households consuming five or more food groups than those consuming four or less food groups. Furthermore, children who did not receive home visits after discharge from the outpatient therapeutic program by health workers were 4.2 [AOR = 4.2; 95% CI: 1.92–9.15] times more likely to be wasted when compared to children who received follow-up visits. Finally, readmitted children were 3.98 [AOR = 3.98; 95% CI: 1.43–15.07] times more likely to be wasted than new admissions (Table 6).

**Table 6:** Factors associated with wasting of children aged 6–59 months after 6 months following discharge from OTP in Habro Woreda, East Ethiopia 2020 (n = 306).

**Variables**	**Category**	**Wasted**		**COR (95%CI)**	**AOR (95% CI)**
		**Yes** = **84**	**No** = **216**		
		***N*** **(%)**	***N*** **(%)**		
Gender of HH head	Men	76 (25.8%)	201 (74.2%)	1	1
	Women	14 (48.2%)	15 (51.8)	2.68 (1.32,5.83)	0.39 (0.11,1.42)
Educational status of the mother	Illiterate	74 (34.1%)	143 (65.9%)	2.78 (1.44,5.38)	3.46 (1.14,10.47)^*^
	Literate	16 (18.0%)	73 (82.0%)	1	1
Availability of latrine	No	49 (44.1%)	62 (65.9%)	3.47 (2.05,5.87)	2.91 (1.33,6.37)^*^
	Yes	41 (21.0%)	154 (79.0%)	1	1
Source of drinking water	Unimproved	39 (42.8%)	52 (57.2%)	2.73 (1.68,4.64)	2.16 (0.94,4.94)
	Improved	48 (22.3%)	167 (77.7%)	1	1
Separate kitchen	No	18 (46.2%)	21 (53.6%)	2.67 (1.33,5.35)	3.42 (1.24,9.45)^*^
	Yes	69 (25.8%)	198 (74.2%)	1	1
Smoking	Yes	73 (34.4%)	139 (65.6%)	3.02 (1.57,5.81)	2.48 (0.99,6.20)
	No	17 (18.1%)	77 (81.9%)	1	1
Household food security	Food insecure	63 (45.7%)	75 (54.3%)	4.98 (2.86,28.68)	4.11 (1.87,9.0)^*^
	Food secure	25 (14.9%)	143 (85.1%)	1	1
Dietary diversity	<4 food groups	79 (36.4%)	138 (63.6%)	5.47 (2.51,11.94)	7.44 (2.58,21.45)^*^
	>5 food groups	10 (11.2%)	79 (88.6%)	1	1
Child illness	Yes	55 (41.0%)	79 (59.0%)	11.75 (6.5,21.26)	6.55 (0.85,15.02)
	No	29 (16.9%)	143 (83.1%)	1′	1
Vitamin A supply for the last 6 months	No	52 (41.3%)	74 (58.7%)	3.31 (1.96,5.60)	1.42 (0.61,3.29)
	Yes	35 (19.4%)	145 (80.6%)	1	1
Home visit after discharge	No	56 (30.6%)	127 (69.4%)	4.85 (2.83,8.33)	4.2 (1.92,9.15)^*^
	Yes	28 (22.8%)	95 (77.2%)	1	1
Admission type	Readmission	14 (41.2%)	20 (58.8%)	3.17 (1.51,6.62)	3.98 (1.43,11.07)^*^
	New	65 (23.9%)	207 (76.1%)	1	1

^*^p-value < 0.05, 1, Reference of the variable; CI, Confidence Interval; COR, Crude Odds Ratio; AOR, Adjusted Odds Ratio; HH, household.

## 4 Discussion

This study was carried out to assess the prevalence and associated factors among children aged 6–59 months after 6 months post-discharge from an outpatient therapeutic program in Habro Woreda, Oromia, Eastern Ethiopia. The result of this study showed that the prevalence of wasting among children aged 6–59 months after 6 months of discharge from the outpatient therapeutic program was 28% (95% CI: 26.5–32.2%). Moreover, the current study revealed that the educational status of the mother, availability of a latrine, separate kitchen, household food insecurity, household dietary diversity, home visit, and admission type were significantly associated with wasting.

According to this study, children born to mothers or caregivers who were illiterate were 3.46 times more likely to experience wasting than their counterparts. This is in line with the studies conducted in Northern Ethiopia (35) and Gurage, Central Ethiopia (20). This is probably because education explicitly enhances the knowledge and awareness of caregivers/mothers to utilize optimal, balanced, and healthy food, which ultimately prevents the likelihood of getting wasted.

In this study, children from households without latrines were significantly associated with wasting. Specifically, the odds of developing wasting among children from households without latrines were 2.91 times higher than their counterparts. This is in agreement with the studies conducted in different settings (4, 19, 22, 31, 36, 37). One plausible explanation could be that those without latrines are explicitly predisposed to diarrheal diseases, which in turn hugely and profoundly affect their nutritional status, ultimately leading to wasting (38–41). Moreover, other sanitation problems related to food are more likely to occur in children who live in households without latrines. More importantly, the lack of latrines might be tied to low socioeconomic status, and limited knowledge and awareness of the importance of having latrines, thereby playing a role in exacerbating problems related to wasting (42, 43).

In the present study, a lack of a separate kitchen was found to be an independent predictor of wasting among children. Accordingly, those children from households who did not have a separate kitchen were 3.42 times more likely to experience wasting than their counterparts. This is in line with the study conducted in West Ethiopia (44). One possible reason could be related to the fact that those households without kitchens are probably of low socioeconomic status, which vividly indicates that the individual households are affected by the problems. A more compelling argument might be that households might utilize solid fuel because of their lower socioeconomic status, which plays a critical role in increasing their likelihood of developing oxidative stress, a condition characterized by an imbalance of free radicals and antioxidants in the body and leads to cell damage (35, 45). Smoking solid fuel is a significant risk factor for respiratory disorders, vascular, and organ diseases owing to its high content of harmful chemicals and reactive oxygen species (ROS). These diseases can lead to depression and lack of appetite for food, which in turn causes wasting among children (35, 45–47). Another plausible explanation could be that solid fuel exposure from cooking and heating may result in overlapping effects and constitute cumulative damage to human health, which clearly also includes wasting (48).

The current study confirmed that children who came from food-insecure households and households that consumed less than or equal to four food groups were more likely to develop wasting than their respective counterparts. This result is supported by the studies conducted in Nepal (49), Tanzania (50), and Ghana (51). Household food insecurity and consumption of low-diversity foods are associated with wasting. The probable cause of food insecurity and lack of diversification of food groups is the low socioeconomic position or low monthly income, which affects households' purchasing power, which in turn reduces access to food (52). Children who consume diverse diets are less likely to be undernourished than those who consume a less diverse diet (53–56). Appropriate education should be given to caregivers of children or mothers on the diversification of food to feed their children and nourish them with a balanced diet.

## 5 Limitations of the study

There might be potential recall bias among respondents when they are answering questions related to past events, and laboratory investigations and related data were not collected in this study. Moreover, being cross-sectional, the study does not address the seasonal variation of child nutritional status or the cause-and-effect relationship between wasting and associated factors, and it uses a single outcome measurement technique. In this study, the variable mid-upper arm circumference (MUAC) at admission, MUAC at discharge, and following child discharge linked or non-supplemental to the feeding program were not included.

## 6 Conclusion

This study showed that the educational status of the mother, availability of a latrine, separate kitchen, household food insecurity, household dietary diversity, home visit, and admission type are significantly associated with the wasting of children after discharge from an outpatient therapeutic program. Therefore, efforts that target these factors should be maximized to reduce the occurrence of wasting among children aged 6–59 months after discharge from the outpatient therapeutic program.

## Data availability statement

The original contributions presented in the study are included in the article/supplementary material, further inquiries can be directed to the corresponding author.

## Ethics statement

Ethical clearance was obtained from the Institutional Health Research Ethics Review Committee (IHRERC) of the College of Health and Medical Sciences, Haramaya University. Informed written/verbal consent was obtained from all subjects and/or the children's parents or caregivers or legal guardian(s). Confidentiality and privacy of every respondent's information were ensured and maintained by using codes instead of the participant's name. Participants were also informed that they have the full right to refuse participation or withdraw at any time from the research. All methods were performed in accordance with relevant guidelines and regulations in the Declaration of Helsinki.

## Author contributions

ST: Conceptualization, Data curation, Formal analysis, Writing – original draft. KR: Methodology, Writing – original draft. HR: Methodology, Writing – review & editing. JN: Conceptualization, Data curation, Writing – original draft. TS: Data curation, Methodology, Writing – review & editing. BA: Conceptualization, Methodology, Writing – original draft. MM: Conceptualization, Methodology, Writing – review & editing. AD: Conceptualization, Methodology, Writing – review & editing. AE: Conceptualization, Methodology, Writing – original draft, Writing – review & editing. IM: Conceptualization, Data curation, Formal analysis, Investigation, Methodology, Supervision, Validation, Writing – original draft, Writing – review & editing.
